# Diverse COVID-19 CT Image-to-Image Translation with Stacked Residual Dropout

**DOI:** 10.3390/bioengineering9110698

**Published:** 2022-11-16

**Authors:** Kin Wai Lee, Renee Ka Yin Chin

**Affiliations:** Faculty of Engineering, Universiti Malaysia Sabah, Kota Kinabalu 88400, Malaysia

**Keywords:** COVID-19, image synthesis, chest computed tomography, generative adversarial networks

## Abstract

Machine learning models are renowned for their high dependency on a large corpus of data in solving real-world problems, including the recent COVID-19 pandemic. In practice, data acquisition is an onerous process, especially in medical applications, due to lack of data availability for newly emerged diseases and privacy concerns. This study introduces a data synthesization framework (sRD-GAN) that generates synthetic COVID-19 CT images using a novel stacked-residual dropout mechanism (sRD). sRD-GAN aims to alleviate the problem of data paucity by generating synthetic lung medical images that contain precise radiographic annotations. The sRD mechanism is designed using a regularization-based strategy to facilitate perceptually significant instance-level diversity without content-style attribute disentanglement. Extensive experiments show that sRD-GAN can generate exceptional perceptual realism on COVID-19 CT images examined by an experiment radiologist, with an outstanding Fréchet Inception Distance (FID) of 58.68 and Learned Perceptual Image Patch Similarity (LPIPS) of 0.1370 on the test set. In a benchmarking experiment, sRD-GAN shows superior performance compared to GAN, CycleGAN, and one-to-one CycleGAN. The encouraging results achieved by sRD-GAN in different clinical cases, such as community-acquired pneumonia CT images and COVID-19 in X-ray images, suggest that the proposed method can be easily extended to other similar image synthetization problems.

## 1. Introduction

Machine learning (ML)-integrated COVID-19 diagnostic systems with medical imaging modalities have shown promising performances in various areas of the healthcare system amid the ongoing COVID-19 pandemic, such as in disease transmission control, patient management, and pathological studies [1,2,3,4].

Although medical imaging-based COVID-19 diagnosis has been reported since the outbreak of the pandemic [5,6,7], large-scale implementation of the medical imaging-based diagnostic system remains in a dilemma due to concern about the risk of exposure to the coronavirus at imaging facilities, strained healthcare systems, and the movement control enforced by local authorities [8]. In addition, the existence of the invariance radiographic features of COVID-19 that are shared among other types of pneumonia hinders the accurate differential diagnosis among these types of pneumonia, including influenza A [9], bacterial pneumonia [10,11], community-acquired pneumonia (CAP) [12,13], and interstitial syndrome [14]. Despite the controversial state regarding the COVID-19 diagnosis with imaging modalities, there is solid evidence that supports radiography approaches in extreme and critical scenarios [15,16,17]; for example, patients who failed the RT-PCR test but have clinical symptoms of COVID-19 or have a history of close contact with COVID-19-infected individuals.

The continuous emergence of COVID-19 variants has aggravated the impaired healthcare system globally [18]. More importantly, the new variants will likely diminish the effectiveness of the existing ML models that were previously trained and tested only on the early COVID-19 image data. This complication is due to the uncertain characteristics of these variants, and their impact on existing ML models remains obscure. The dominant radiographic feature of COVID-19 is ground-glass opacity (GGO), which presents with a grayish color and fiber-like texture that is distributed within the region of the lungs in “Crazy-paving” and “reversed halo sign” patterns [16,17,19]. The GGO abnormalities can vary from large to small patches, and can be difficult to interpret from human observation due to the invariances in radiography characteristics within the pneumonia family [9,10,11,12,13,14] and vast pattern diversity, especially when the abnormalities appear to be subtle and nanoscopic on observation.

The performance of most ML models is generally known to depend on the quantity and quality of the training data. However, medical data or images are exceptionally scarce due to privacy concerns and a lack of data, especially for newly emerged diseases [20,21,22,23]. Thereby, data acquisition plays an all-important role in accelerating the development of feasible machine learning models that may potentially assist the COVID-19 and COVID-19 variants diagnostic systems.

The active research field of generative modeling has advanced the performance of many machine-learning-based image synthetization solutions in a wide range of problems, such as image-to-painting, image colorization, image transfiguration, and semantic map-to-image [24,25,26,27]. Image synthesization via generative modeling is emerging as a powerful solution to data paucity in many data-driven machine learning models for medical imaging applications such as image synthetization [20,21,22,23,28], segmentation [29,30], super-resolution [31], and classification [32].

The rapid advancement of I2I translation has facilitated many state-of-the-art image synthesization approaches that continue to break through the limitations of conventional methods in areas of super resolutions [33,34], unpaired I2I translation [21,35,36], multimodal image translation [37,38,39], and fine-grained translation [40,41]. Very recently, various GANs have been adopted to generate synthetic COVID-19 CT [21,28,42] and X-ray images [22,23], which are formulated as a problem of I2I translation. From the literature, the motivation for the synthetization of COVID-19 imaging modalities is mainly derived from the scarcity of image data for high-performance and robust machine-learning models developed for COVID-19 detection and classification tasks [21,22,42].

The synthetization of COVID-19 image data in the field of I2I translation can be interpreted as a transfer of features, where the image translator learns the image mapping between non-COVID-19 and COVID-19 data distributions and distinguishes the classes with the unique feature attributes that belong to each class. This powerful learning algorithm encourages the synthetic images to manifest new instances that are consistent with the real data distribution. As a result, generative data augmentation facilitated by I2I translation is superior to the conventional data augmentation approaches that are generally known to suffer from data diversity [43].

From the literature, existing COVID-19 image data synthetization approaches have shown encouraging performances in different problem settings, which can be divided into paired and unpaired image translations. The paired translation aims to replicate the same style features based on a predefined descriptor, such as semantic maps. In contrast, unpaired image translation learns to generate style attributes based on the knowledge obtained without supervision. For instance, paired image translation is explored with the pix2pix GAN model to generate high-quality COVID-19 CT images from semantic layout maps that contain precise annotations of the GGO features within the lung regions [28]. Alternatively, image pairs can be generated using elastic registration algorithms to register features of GGO on non-COVID-19 CT images [42]. Although paired image translation approaches demonstrated excellent performance in image quality, acquisition and curation of large numbers of image pairs are challenging considering the time and effort needed to create the aligned image pairs. Moreover, paired image translation approaches lack feature diversity because they focus only on replicating the GGO features and do not generate new feature instances [28,42].

Recent studies have shown that synthetic COVID-19 chest X-rays can be generated from randomly distributed noise vectors using the auxiliary classifier GAN (ACGAN) [22]. Unlike conventional GANs, the generator of the ACGAN generates the output images from the input noise distribution and the class label of the target distribution. The discriminator receives the generated images and predicts the probability that the image belongs to a specific class and that the input image is real. The original ACGAN study claimed that adding class labels as an input can improve the stability of the training dynamics and enhance the quality of the synthetic images [44]. Although ACGAN does not rely on image pair supervision, in image synthesization it is inevitably challenging to generate high-frequency and detailed structures from noise distribution. Consequently, the GGO features presented in lower resolutions can be misinterpreted as bronchi, bronchioles, or other unrelated abnormalities.

CycleGAN is another exciting extension of GANs, and is trained on unpaired images. It encourages the outputs of the reverse generator to be similar to the input of the forward generator, which forms a cycle-consistency constraint that regularizes the bidirectional image translation dynamics and enhances the quality of the synthetic images [45]. The attractive property of the CycleGAN training algorithm essentially addresses the problem of low image-quality synthesization and the requirement of image pair supervision. CycleGAN has demonstrated promising results in high-quality COVID-19 CT image synthesization from lung cancer images [21]. The authors of the study noted that the image translation takes advantage of the existing annotations from the lung cancer nodules of the lung cancer images to generate plausible features of COVID-19 around the location of the nodules. A similar approach was also found to adapt well to COVID-19 chest X-ray images, and was intended to reduce the class imbalance distribution of COVID-19 X-ray images compared to CAP and health control in a COVID-19 classification task [23].

Most existing COVID-19 imaging modality synthetization approaches generate deterministic outputs, which means only a single output can be generated from every unique input [21,22,23,28,42]. This is a considerable drawback for data augmentation due to the limited image diversity from the deterministic setting of the GANs. By comparison, most multimodal GAN frameworks aim to maximize perceptual diversity via disentangled representations, which results in significant perceptual differences [38,39]. As such, image translation for medical images that relies on subtle and detailed visual descriptions of the radiographic findings is more appropriately formulated using a fine-grained feature transfer approach. In particular, fine-grained image translation aims to transfer only the fine contexture details of the images. However, fine-grained feature transfer approaches lack image quality preservation due to the sizeable geometrical deformation and dependency on Variational Autoencoders (VAEs), which are prone to generate blurry images [40,41].

The utilization of regularization techniques to encourage image diversity has been investigated previously using Gaussian noise addition and dropout layers in U-net architecture [46]. However, the stochasticity induced by the proposed regularization strategy did not generate perceptually significant structural variances in the image transformation tasks. In recent work, Yang et al. regulated the generator using a maximization objective conditioned on two randomly sampled noise latent codes [47]. Although the proposed method was explicitly designed for cGAN algorithms, the capability of the method in facilitating fine-grained feature diversity and its effectiveness in unsupervised training algorithms remain obscure.

In essence, based on the literature survey, existing works for the synthesization of fine-grained and multimodal outputs for medical imaging are limited. Synthetic data hold great value as supplementing data for AI-based medical imaging applications where the diagnostic models can benefit from diverse training data without conducting actual imaging procedures.

This paper presents an image synthesization framework that generates high-quality and realistic synthetic COVID-19 CT images, which can be a practical solution to data paucity and supplement the development of ML models. The image synthesization framework is built on an image-to-image (I2I) translation framework that can translate radiological features of GGO on non-COVID-19 chest CT images. In this study, sRD-GAN is used. Unlike existing COVID-19 synthetization methods that generate deterministic outputs, sRD-GAN can generate instance-diverse outputs from a single input without relying on prior distributions or auxiliary conditions.

The non-COVID19-to-COVID19 CT I2I translation is formulated as a problem of image transfiguration, which involves transferring style features across the image domain with highly similar global structures, such as the horse-to-zebra or orange-to-apple translation [45]. However, sRD-GAN has fundamental differences compared to general-purpose I2I translation problems due to three vital criteria:

1. Diverse style attributes of GGO features: the radiographic features of COVID-19 on chest CT contain the formation of GGO in various shapes and sizes. This setting is in contrast with the general-purpose unimodal I2I approaches, where the style attributes are easily identified with a single representation, such as black and white lines (zebra), brown color region (horse), and orange color regions (orange) [45]. Therefore, the non-COVID19-to-COVID19 translation is a more challenging task and requires translating the style attributes presented in different patterns and locations within the region of the lungs.
2. Requirement of high-frequency structures: in the general-purpose I2I translation setting, minor and detailed variations of style attributes generated on the outputs usually do not affect the global representation of the image due to the low requirement for high-frequency representation. However, the natural attributes of the GGO features are represented in high-frequency details and manifested in different shapes, locations, and sizes [16,17,19]. Therefore, these detailed GGO patterns generated within the lung parenchyma are essential in deciding the perceptual realism of the synthetic COVID-19 CT images.
3. High tolerance of texture artifacts: ideal image translation generates images containing no traits of style attributes that overlap with the source domain, also known as style artifacts. Texture or style artifacts refer to the translation error of style attributes, which cause overlapping style attributes that belong to both image domains. Such concern is negligible for non-COVID-19-to-COVID-19 translation because the style attributes of the images from both domains share similar radiographic representations. As such, overlapping style attributes in style artifacts are hardly visible, and do not affect the general realistic representation of the synthetic outputs.

sRD-GAN is incorporated with the proposed stacked residual dropout (sRD) mechanism, which systematically uses dropout regularization to facilitate latent space stochasticity and generate perceptually significant image differences. Since the proposed method differs from most multimodal or diverse image translation methods based on disentangled learning, it can generate instance-diverse outputs from the same input without depending on prior distribution or auxiliary conditions. In addition, an adaptive pixel consistency loss is also proposed to reduce the translational noises and enhance the perceptual realism of the synthetic outputs.

The contributions of this paper can be summarized as: (1) a novel diverse I2I translation framework (sRD-GAN) is designed to facilitate COVID-19 CT image synthesization with instance-level diversity; (2) an adaptive pixel consistency is introduced to improve the perceptual quality of the synthetic COVID-19 CT images; and (3) an in-depth investigation is conducted of the performance of various existing GAN models on the newly defined COVID-19 image translation task.

## 2. Material and Methods

### 2.1. Datasets

The COVID-19 and non-COID-19 CT images were provided by the Union and Liyuan Hospitals affiliated with Huazhong University of Science and Technology; the data contain substantial CT data with precise metainformation such as SARS-CoV-2 nucleic acids outcomes, CT outcome, morbidity, and mortality. To maximize information diversity and quality, 100 subjects (patients) were curated from the iCTCF dataset acquisition platform [4], where each subject was manually inspected to ensure the positive COVID-19 CT images contain a substantial magnitude of GGO features manifested in different chest anatomies. Similarly, 200 subjects not infected with COVID-19 were also curated with an emphasis on the diversity of chest structure. From the same platform, 9575 chest CT images without clinical conditions and diseases from a segregated dataset (HUST-19 dataset) [4] were acquired for testing purposes.

In addition, 416 COVID-19 subjects and 412 CAP clinical subjects were also acquired from [48] to enhance the generalizability of the image synthesizer, where the images were provided by the Xiangyang Central Hospital and Xiangyang No. 1 People’s Hospital, Hubei, China. Furthermore, 13,408 COVID-19 and non-COVID-19 chest X-ray images were acquired from [49] to evaluate the adaptivity of the image synthesizer in different imaging modalities. The information on the datasets is summarized in Table 1.

### 2.2. Stacked Residual Dropout (sRD) Mechanism

#### 2.2.1. Assumption

Multimodal I2I translation generates diverse outputs using disentangle learning that assumes images can be disentangled into a shared content latent space *c*_i_ ∈ *C*, which represents the invariance features from both image domains, and a style latent space *s*_i_ ∈ *S*, which represents the distinctive class attributes of the image domains. However, in the problem of non-COVID19-to-COVID19 I2I translation, the features of GGO represented on CT images are complex and vary with different patients. Thus, this study assumes that the images from the image domains *x*_i_ ∈ *X*, non-COVID-19 CT images, and *y*_i_ ∈ *Y*, COVID-19 CT images, share a large content latent space *c*_i_ ∈ *C* and the unique radiographic features of GGO are assumed to be *s*_i_ ∈ *S*. Finally, the non-COVID19-to-COVID19 CT I2I translation is formulated as is *y*_i_′ = *G*(*x*_i_), where *y*_i_′ = *c*_i_ × *s*_i_, so that *Y*′ ≈ *Y*, where *Y*′ is the synthetic COVID-19 CT distributions.

The dropout layers incorporated within the residual blocks facilitate latent space stochasticity, which is induced directly in the bottleneck model of the image transformation network. As such, the arbitrary style attribute generated with the stacks of residual dropout is formulated as *s*_i_′ = *p*_i_ × *s*_i_, where *p*_i_ is the dropout variable representing an arbitrary dropout state based on the predefined dropout rate [50].

#### 2.2.2. Building Block of Residual Connections

**Figure 1:**
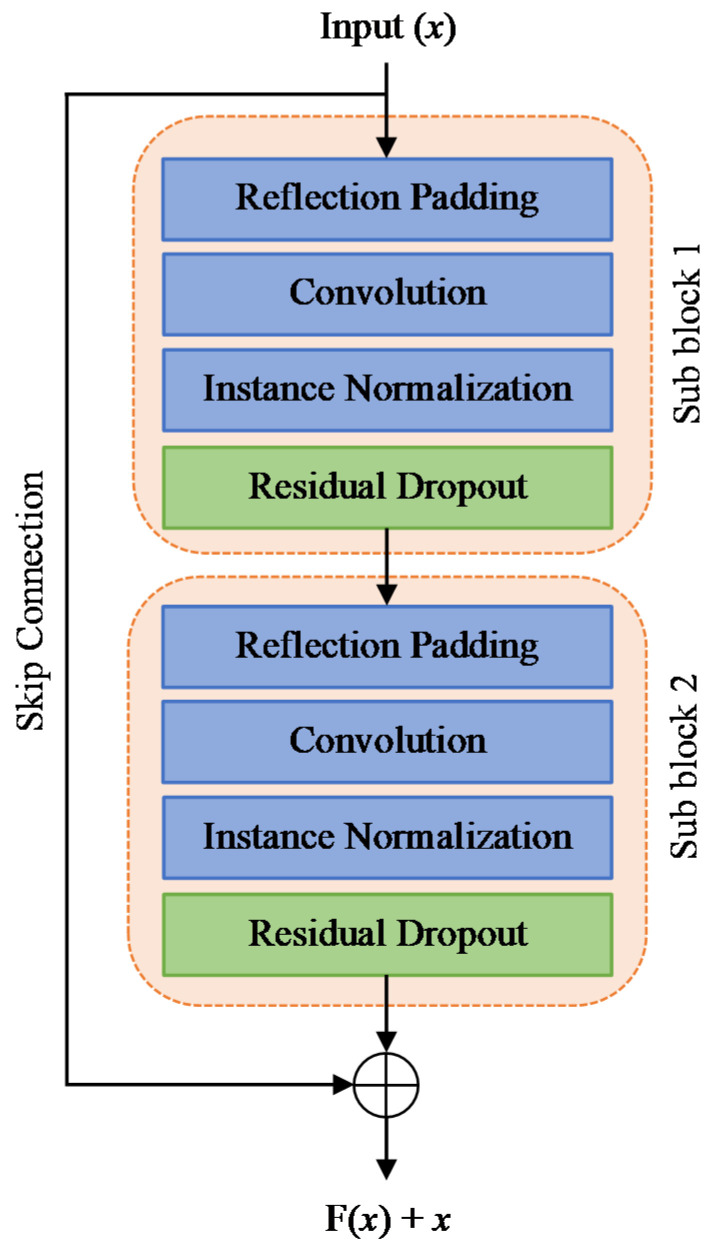
A single residual block of the image transformation network. The residual dropout regularization layer is added after the instance normalization layer at each sub-residual block.

The multiple blocks of residual connections within the image transformation network form a deep residual convolutional neural network (CNN) that learns a single direction image mapping *G*(*x*) → *y*′:

(1) $$y_{1}^{\prime }=p_{1}\times res_{1}\left(x_{1}\right)$$

(2) $$y_{2}^{\prime }=p_{2}\times res_{2}\left(y_{1}^{\prime }\right)$$

where *res*_i_() denotes the sub-residual block in a single residual block, *x*_1_ is any arbitrary input instance from the previous layer, *y*_1_′ is the output instance for the first sub-residual block, *y*_2_′ is the output instance for the second sub-residual block, and *p*_i_ represents the dropout variable based on the predefined dropout rate. Figure 1 shows that each residual block contains two identical sub-blocks with residual dropout layers after the instance normalization layers. The input instance to the first sub-residual block is concatenated with the output of the second residual sub-block via the skip connection [51]. Activation layers are omitted for simplicity.

#### 2.2.3. Two-Mode Structure of sRD Mechanism

The sRD mechanism operates in two different modes and the configurations of the residual dropout layers of the image transformation network change between the modes. In the training mode, the image transformation network is trained to learn the image mapping *G*(*x*) → *y*′ in a highly stochastic environment, where *x* is from the non-COVID-19 CT domain and *y*′ is the synthetic COVID-19 CT image generated by *G*. The output instance of the single residual block is:

(3) $$y_{1}=p_{1}RES_{1}\left(x\right)+x$$

where *RES*_i_() denotes the main residual block in a single residual block, and *p*_i_ denotes the predefined dropout variable for the residual blocks. The outputs for the subsequence residual blocks are:

(4) $$y_{2}=p_{2}RES_{2}\left(y_{1}\right)+y_{1}$$

(5) $$y_{3}=p_{3}RES_{3}\left(y_{2}\right)+y_{2}$$

(6) $$y_{4}=p_{4}RES_{4}\left(y_{3}\right)+y_{3}$$

…

(7) $$y_{9}=p_{9}RES_{9}\left(y_{8}\right)+y_{8}$$

**Figure 2:**
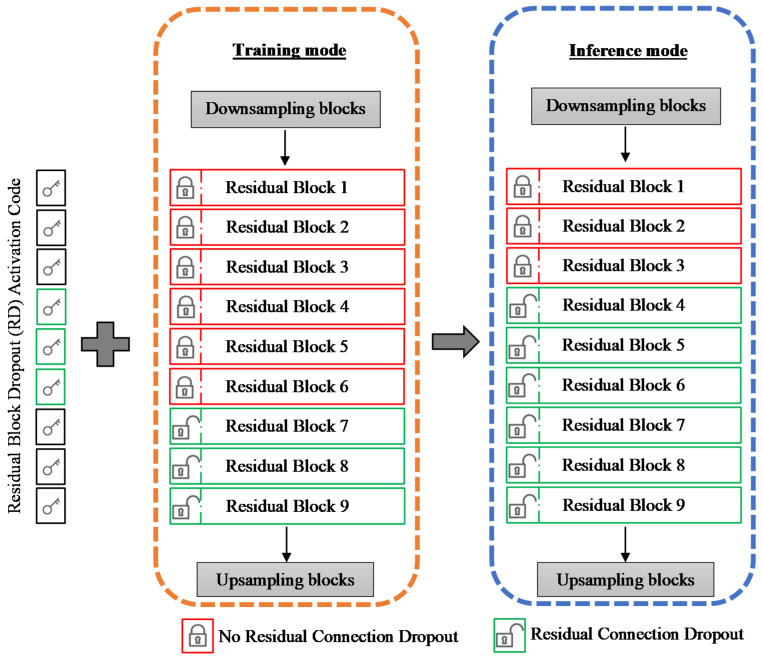
Image transformation network incorporated with the residual dropout mechanism in the training mode and the inference mode. The RD-activation code illustrates the reconfiguring of the residual dropout at the inference mode to amplify the latent space stochasticity without any model retraining.

Therefore, the image transformation network can be formulated as a function of *x* such that:

(8) $$y_{act}=F\left(x\right)$$

where *y_act_* denotes the activation output from the final residual block, and *x* is the final down-sampled input to the image transformation network. The padding, normalization, and activation layers are excluded from the formula for simplicity. Figure 2 illustrates the configuration of the image transformation network in both training and inference modes.

In the inference mode, the residual dropout (RD)-activation code is applied to readjust the stochasticity setting of the trained generator model without fine-tuning or training. The RD-activation code is a user-defined instruction that activates or deactivates the incorporated dropout layers within the residual blocks. The rationale of the stochasticity setting readjustment posterior to the completion of training is to amplify the magnitude of the latent space stochasticity relative to its initial state achieved in the training mode. As such, a higher magnitude of latent space stochasticity can be achieved without readjusting the parameter of the bottleneck model.

### 2.3. Stacked Residual Dropout GAN Framework (sRD-GAN)

#### 2.3.1. Overview of sRD-GAN

**Figure 3:**
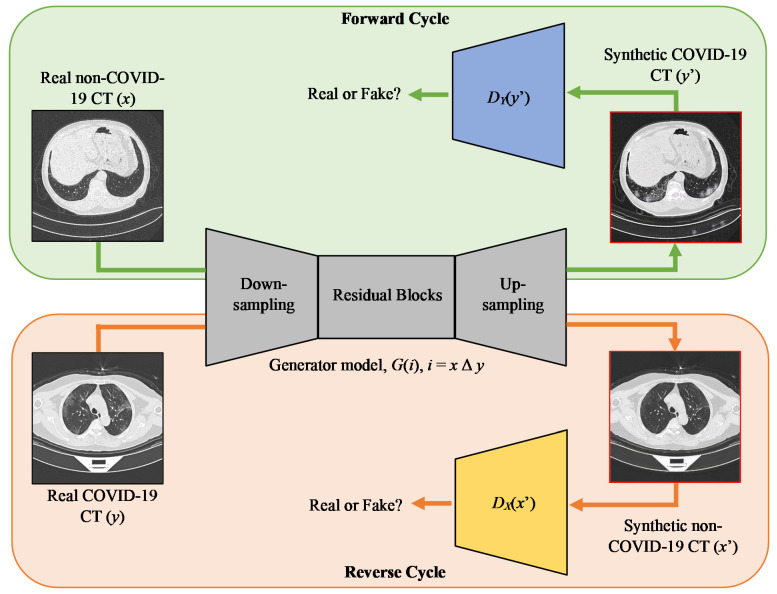
Overview of the sRD-GAN framework. Bidirectional image mapping uses only a single generator model and two different discriminative models for both mapping directions.

The sRD-GAN framework consists of one generator model and two discriminator models. The generator model learns the bidirectional image mapping from *G*(*x*) → *y*′ and *G*(*y*) → *x*′, while the discriminator models predict the outputs from respective mapping directions. The sRD-GAN framework is illustrated in Figure 3.

Unlike CycleGAN, sRD-GAN consists of only one generator model to generate synthetic outputs from both image domains, assuming the generator model exerts a self-inverse property [36]. This also assumes that a single generator model is sufficient to learn the bidirectional image mapping across two image domains. Although the single generator structure does not benefit the time efficiency for the same image batch size compared to the concurrent CycleGAN frameworks [36,45], it doubles the learning frequency of the single generator model, which has been proven to improve the quality of the synthetic images [36].

#### 2.3.2. Models

The basic architecture of the generator and discriminator models was designed based on [46,52], respectively. The generator model comprises three different types of blocks. In this work, it consists of two down-sampling blocks, nine residual blocks, and two up-sampling blocks. The discriminator models are PatchGAN models that map inputs to 2D arrays, which indicates whether the synthetic outputs are fake or real [46]. Details of the model architectures are included in Table A1 and Table A2 in Appendix A of this paper.

### 2.4. Lost Function and Formulation

#### 2.4.1. Adaptive Pixel Consistency Loss

A new adaptive pixel consistency loss is introduced to enhance the perceptual realism of the synthetic COVID-19 CT images for improvement of the perceptual realism of the images. The pixel consistency loss is utilized as a mapping constraint to exert a strong correlation between the input image and its corresponding output image by encouraging them to be similar via the mean absolute error.

In contrast to conventional loss functions, for which importance is controlled by constant values, the proposed adaptive setting of the pixel consistency loss function changes the importance of the loss based on the generator loss at each training step. Therefore, a smaller error resulting from the generator will correspond to a smaller weight update. The pixel consistency loss for both image mapping is defined as:

(9) $$L_{pixel}^{x}(G)=E_{x∼Pdata(x)}[∥G(x)-x{∥}_{1}]$$

(10) $$L_{pixel}^{y}(G)=E_{y∼Pdata(y)}[∥G(y)-y{∥}_{1}]$$

The adaptive setting of the pixel consistency loss is defined by:

(11) $$\lambda_{pixel}^{x}=\min\left[\log\left(D_{Y}(y)\right)+\log\left(1-D_{Y}(G(x))\right)\right]$$

(12) $$\lambda_{pixel}^{y}=\min\left[\log\left(D_{X}(x)\right)+\log\left(1-D_{X}(G(y))\right)\right]$$

#### 2.4.2. Adversarial Loss 

Adversarial training aims to learn the mapping function in a min-max theory [53], where the generator aims to generate outputs that are ideally similar to the distribution of the target domain. In contrast, the discriminator aims to distinguish the fake and good samples generated by the generator model. The generator and discriminator exert an adversary relationship, where a win in the discriminator would result in a loss in the generator, and receive a penalty in the form of weight updates accordingly. The adversarial loss for the forward and backward mapping direction based on one-to-one CycleGAN [36] is formulated as follows:

(13) $$L_{GAN}\left(G,D_{Y},X,Y\right)=E_{y∼Pdata(y)}\left[\log D_{Y}(y)\right]+E_{x∼Pdata(x)}\left[\log D_{Y}\left(1-D_{Y}(G(x))\right)\right]$$

(14) $$L_{GAN}\left(G,D_{X},X,Y\right)=E_{x∼Pdata(x)}\left[\log D_{X}(x)\right]+E_{y∼Pdata(y)}\left[\log D_{X}\left(1-D_{X}(G(y))\right)\right]$$

#### 2.4.3. Cycle Consistency Loss

Similar to CycleGAN and other concurrent models, cycle consistency loss reduces the space of possible image mappings in many-to-many I2I translation problems [32,35,36]. The cycle consistency loss is computed as a mean absolute error between the outputs of the reverse generator to the input of the forward generator, which enforces a one-to-one mapping between the image domains in separated latent spaces. Since only one generator model is used in sRD-GAN, the cycle consistency loss is computed from the output images that are generated from the same generator *G*. The cycle consistency loss is defined as:

(15) $$L_{cycle}^{x}\left(G\right)=E_{x∼Pdata\left(x\right)}\left[∥G\left(G\left(x\right)\right)-x{∥}_{1}\right]$$

(16) $$L_{cycle}^{y}\left(G\right)=E_{y∼Pdata\left(y\right)}\left[∥G\left(G\left(y\right)\right)-y{∥}_{1}\right]$$

#### 2.4.4. Identity Loss

The identity loss is effective in preserving the color composition between the input and its corresponding output, based on the original CycleGAN study [45]. The identity loss function is modified in this work since only one generator is used in the sRD-GAN framework. The identity loss is computed between *X*′ and *Y* for forward mapping and *Y*′ and *X* for reverse mapping. The formulation of the identity loss is given by:

(17) $$L_{id}^{y}\left(G\right)=E_{y∼Pdata\left(y\right)}\left[∥G\left(y\right)-y{∥}_{1}\right]$$

(18) $$L_{id}^{x}\left(G\right)=E_{x∼Pdata\left(x\right)}\left[∥G\left(x\right)-x{∥}_{1}\right]$$

#### 2.4.5. Full Objective Functions

The final objective function of the sRD-GAN for *G*(*x*) → *y*’ is:

(19) $$L\left(G,D_{Y}\right)=L_{GAN}\left(G,D_{Y},X,Y\right)+\lambda_{cycle}^{x}L_{cycle}^{x}(G)+\lambda_{pixel}^{x}L_{pixel}^{x}(G)+\lambda_{id}^{y}L_{id}^{y}(G)$$

and the final objective function for the reverse cycle *G*(*y*) → *x*’ is:

(20) $$L\left(G,D_{X}\right)=L_{GAN}\left(G,D_{X},X,Y\right)+\lambda_{cycle}^{y}L_{cycle}^{y}(G)+\lambda_{pixel}^{y}L_{pixel}^{y}(G)+\lambda_{id}^{x}L_{id}^{x}(G)$$

### 2.5. Experiment Setup

#### 2.5.1. Implementation

For the experiment, the curated dataset pool was divided into training and testing datasets without overlapping. In particular, one image was manually selected from 100 explicitly segregated patients from [4] as the test set. The axial slice of the CT data was manual selected to ensure the test images are diverse in terms of chest and lung structures since the adjacent CT slices can be close to identical. The images for training were randomly selected from the remaining data pool, creating a training dataset of 800 images that consists of 400 images from the two image domains of COVID-19 and non-COVID-19. In addition, 3000 non-COVID-19 CT images were randomly selected from 9575 images of the HUST-19 dataset, which were used as an additional test set to evaluate the generalizability of sRD-GAN.

Following [21,45], the batch size for training the sRD-GAN is set at one, and a complete training cycle requires 400 training steps. Each training step uses one image from the respective image domain to form a training pair (*x*_i_, *y*_i_). Unlike the paired training method, the images need not be aligned and are shuffled randomly during the preprocessing stage. Due to the graphics memory limitation, the training images are resized to a spatial dimension of 256 × 256. During the inference time, the test images are kept at the high-resolution setting of 512 × 512. The images for training and interface are color scaled with three RGB color channels.

In the preliminary experiment, it was observed that the quality of the images does not improve further after 40–50 epochs. Therefore, the learning rate was fixed at 0.0002 for the first 50 epochs and exponentially decayed for the following ten epochs. Similar to CycleGAN [45] and one-to-one CycleGAN [36], the Adam optimizer was used as an optimizer with a beta_1 value of 0.5 and beta_2 of 0.999. The weights of each layer and instance normalization were initialized from a Gaussian distribution *N*(0, 0.02).

The training procedure for every training step is as follows:

(1) *x*_i_ is fed as input to learn the forward mapping *G*(*x*_i_) → *y*_i_′, and backpropagate *G* and *D_Y_*.
(2) *y*_i_ is fed as input to learn the reverse mapping *G*(*y*_i_) → *x*_i_′, and backpropagate *G* and *D_X_*.

#### 2.5.2. Software and Hardware Specification

All simulation works, including data processing, neural network training, testing, and analyses, were computed in PyCharm IDE Community Edition 2020.2.1 × 64 with Python 3.6. All simulations were performed on a GPU environment for more efficient computation, with CUDA version 10.1, Tensorflow-gpu 2.2.0, and CudDNN version 8.0.2. The hardware resources included a 240 Gb solid state drive memory and NVIDIA GTA 950M as the graphics processing unit. A single training step took four seconds, and the model required 106.67 h for training completion (60 epochs, 400 steps each) and one second to generate one synthetic image in the inference mode.

### 2.6. Performance Evaluation

#### 2.6.1. Radiologist Examination

The perceptual realism of the synthetic COVID-19 CT images was evaluated using the Visual Turing Test performed by a radiologist with ten years of experience in General Clinical Radiology and Pediatric Radiology. In this test, 50 synthetic COVID-19 CT, 30 real COVID-19 CT images, and 20 real non-COVID-19 CT images were included. Of the 50 synthetic images, 20 synthetic images containing visible style artifacts were intentionally included in the test for comparison. The radiologist was informed that the images contained real and synthetic images, but was not told which were real and which were synthetic images for blind evaluation. The Visual Turing Test results are illustrated using confusion matrixes that separate the real COVID-19, synthetic COVID-19, and real non-COVID-19 classes.

#### 2.6.2. Learned Perceptual Image Patch Similarity (LPIPS)

The Learned Perceptual Image Patch Similarity (LPIPS) distance quantifies the image diversity by computing the distance between image batches, which are the activations generated by the pre-trained AlexNet model [54]. In this study, the LPIPS distance quantifies (1) instance diversity between synthetic outputs generated from the same inputs and (2) significance of the synthetic COVID-19 CT images, representing the perceptual differences between synthetic COVID-19 CT images and their corresponding real non-COVID-19 CT image inputs. For instance-diversity, larger LPIPS distances signify that the images are perceptually more different. For the significance of the synthetic features, larger LPIPS distances indicate more significant features presented on the synthetic images.

#### 2.6.3. Fréchet Inception Distance (FID)

The FID score is generally used to measure the quality of the synthetic outputs generated by GAN models [55]. It compares the statistical measurement from real and synthetic data without considering the image class. Using the pre-trained Inception V3 model, the activations of the real and synthetic images are computed as a multivariate Gaussian using the covariance and mean of both types of images. Then, the distance between the two distributions is calculated as the FID metric, where lower scores imply that the synthetic image batch and the real image batch are statistically more similar, which signifies higher quality.

#### 2.6.4. Statistical Analysis

In this study, the Uniform Manifold Approximation and Projection (UMAP) [56] is computed to identify the correlations between the three distributions, including the real COVID-19, real non-COVID-19, and synthetic COVID-19 CT images generated by sRD-GAN. For this purpose, a pre-trained COVID-19 detection network [57] is used to extract the feature maps of the images to generate the high dimensional representation of these data distributions using the UMAP algorithm. Clusters that are closer to each other signify that the instances of the data belonging to these clusters are more similar. Additionally, the structural similarity between the synthetic and real COVID-19 CT images is also analyzed using a histogram illustration between the pixel distributions belonging to different image classes. To further validate the synthetic data generated by sRD-GAM, the Gradient Class Activation Map (Grad-CAM) is computed to identify the saliency response of the COVID-19 detection model on the data distributions using the feature maps generated by the pre-trained COVID-19 detection model [57]. The saliency maps generated by the COVID-19 detection model that was trained with only real data can provide essential insights into the coherences between the synthetic and real COVID-19 CT images and discriminative behavior against real non-COVID-19 CT images.

## 3. Result and Discussion

In this section, extensive experiments and results are discussed. Firstly, the effect of latent space stochasticity amplification facilitated by the two-mode mechanism of the sRD-GAN is discussed in Section 3.1. Next, the performance of various sRD mechanism designs in inference mode is discussed in Section 3.2, which aims to describe the characterization of the sRD regularization in different dimensionalities and magnitudes of the dropout regularization. Section 3.3 justifies the qualitative evaluation of the synthetic COVID-19 CT images with respect to the image perceptual realism. Lastly, the effectiveness of the proposed adaptive pixel consistency loss function in facilitating effective noise reduction for I2I translation is discussed in Section 3.4.

### 3.1. sRD-GAN in Training and Inference Modes

The sRD regularization is operational in two different modes, where the models are initially trained with a smaller magnitude of residual dropout in training mode and increase the magnitude of the residual dropout for inference without retraining the models. As such, the image instance diversity of the synthetic images generated in training and inference modes can be compared to characterize the amplification effect of the residual dropout facilitated by the duo training-inference mechanism.

In training mode, six variations of sRD regularization are considered, where each variation contains a different number of residual blocks with residual dropout (RD-blocks). From the first to the sixth variation, the increment in the number of RD-blocks begins from the last residual block until the sixth residual block. The illustration of the image transformation network is explained in Figure 2.

The dropout rate is fixed at 0.5 for all RD-blocks in this experiment. In addition, the residual dropout is not activated for the first three residual blocks for all variations of sRD regularization for training in this experiment. In the inference mode, residual dropout is activated for all the residual blocks of the bottleneck model at a fixed dropout rate of 0.5 for all the six sRD-GAN variations. It is important to emphasize that model retraining and fine-tuning are not required for this purpose.

**Figure 4:**
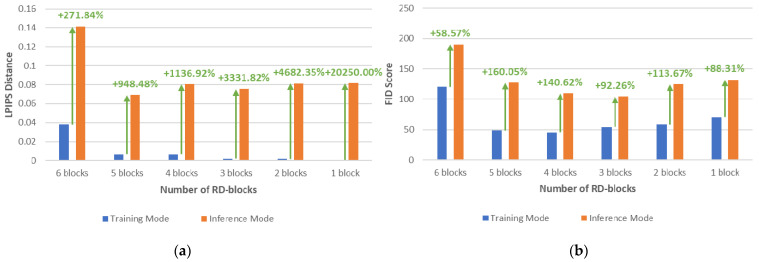
(**a**) LPIPS and (**b**) FID metrics of the synthetic images generated in training and inference modes.

Image instance diversity is evaluated by measuring the LPIPS distance between five output image batches generated from the same input batch, where one reference batch is selected from the five image batches. Figure 4 shows the LPIPS distance and the FID scores of the synthetic images generated with six variations of sRD regularization in training and inference modes. The amplification of the latent space stochasticity facilitated by the two-mode mechanism shows a remarkable increase in instance diversity for all variations of sRD regularization, with the highest increase in LPIPS distance being 20250% between training and inference modes for one RD-block variation.

**Figure 5:**
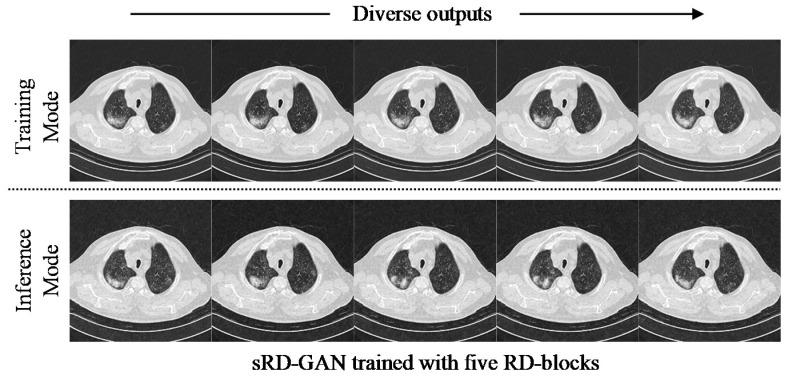
Synthetic COVID-19 CT images generated in training and inference modes, where the diverse outputs generated in inference mode show more apparent differences between the synthetic instances.

Despite the lowest percentage increase of 271.84% by the six RD-block variation, the model achieved the overall highest LPIPS distance of 0.1413, approximately two-fold higher than other variations. Figure 5 shows the perceptual difference output images generated in training and inference modes, where the instance diversity of the synthetic images generated in the inference mode is more significant compared to the images generated in the training mode. Specifically, the GGO instances manifested on the synthetic images generated in inference mode are diverse in shapes and sizes, whereas the GGO instances manifested in training mode are relatively smaller in magnitude.

**Figure 6:**
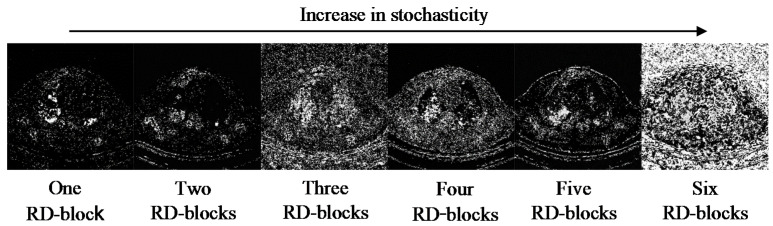
Image difference between a reference output and a second output generated for different numbers of RD-blocks in training mode. The six RD-blocks variation shows the largest magnitude of image difference.

Despite the significantly lower LPIPS distance yielded by the synthetic images in training mode compared to inference mode, the sRD regularization is capable of facilitating sufficient latent space stochasticity that yields a visible image difference, where its magnitude increases with greater numbers of RD blocks, as shown in Figure 6. However, the ample space of latent stochasticity induced in the inference mode negatively impacted the perceptual quality of the synthetic images, where all sRD regularization variations score higher FID in inference mode compared to training mode, as demonstrated in Figure 4. In particular, the highest increase in FID score due to the amplification of latent space stochasticity is observed to be 160.05% by the five RD-block variation, and the largest FID score achieved in the inference mode is found to be 190.57 by the six RD-block variation. Nonetheless, the degradation of perceptual quality of the synthetic images is relatively smaller compared to the significant boost in instance diversity. Moreover, the differences in the instance-diverse outputs and the differences in perceptual quality of the synthetic images generated with different variations of sRD regularization are hardly noticeable based on human observation, except for the six RD-block variation. In addition, Figure 6 also shows a drastic image difference with large patches of noise that encompass the entire image observed in the six RD-block variation compared to other variations. This suggests that training with latent space stochasticity can negatively affect the training process if the magnitude is not capped.

In essence, the key success of the latent space stochasticity amplification that significantly boosts the instance diversity without causing image occlusions or blurry effects is contributed by achieving training stability in a stochastic environment. In addition, a negative correlation between instance diversity and perceptual quality can be established due to the consistent trade-off behavior observed between the two qualities. A possible explanation for this is that the expanded space stochasticity has inevitably led to a more unconstrained space of image mapping, which likely increases the magnitude of noise artifacts, leading to quality degradation.

In addition, the non-linear relationship between the number of RD-blocks and the quality of the synthetic images suggests that the difference in latent stochasticity induced by the varying number of RD-blocks is insufficient to cause significant impacts on the perceptual quality of the output images. In contrast, the high stochastic nature of the learning dynamics in GANs contributes to a more dominant effect on the quality of the images.

### 3.2. Impact of Different RD-Block Designs

The comparison of the images generated with varying numbers of RD-blocks explains the impact of the number of RD-blocks on the qualities of the synthetic images, as discussed in Section 3.1. This section goes presents an in-depth investigation of the impact of various designs of the sRD mechanism on synthetic images. Since the stochasticity setting facilitated by the sRD mechanism is formulated as a function of residual dropout, the magnitude of the stochasticity can be characterized based on the dropout rate, latent depth, and the stacked residual dropout.

#### 3.2.1. Residual Dropout Rate

**Figure 7:**
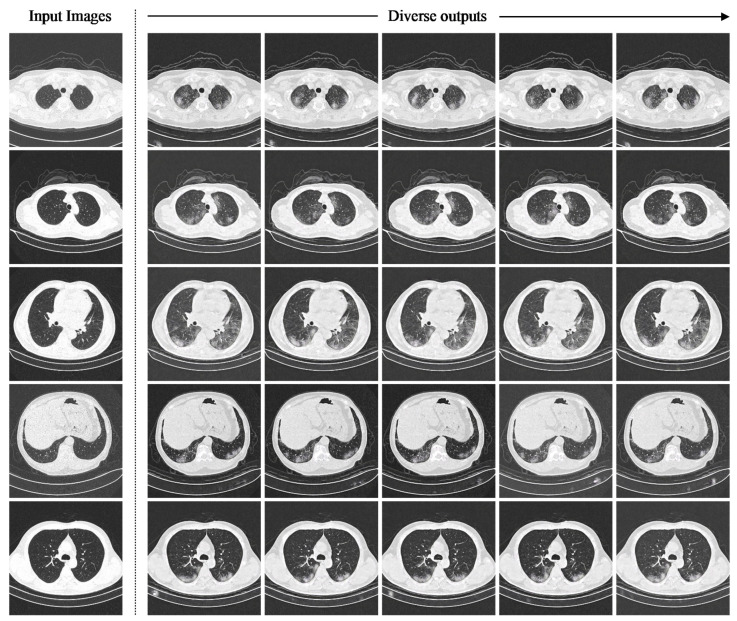
Instance-diverse synthetic images generated from the sRD-GAN with light residual dropout using the same non-COVID-19 inputs in inference mode (0.2 dropout rate). Fine-grained image diversity is consistently observed on the synthetic features of ground-glass opacities (GGOs) generated within the region of the lungs. Other samples are included in Dataset S2.

The impact of the residual dropout rate on the instance diversity is demonstrated by systematically modifying the residual dropout rate of the sRD-GAN in training and inference modes. To facilitate high instance diversity with the preservation of good-quality images, a new variation of sRD-GAN was trained with six RD-blocks with a light residual dropout setting. The only difference between the sRD-GAN with the light residual dropout setting and the previously discussed sRD-GAN, which was also trained with six RD-blocks, is the reduction in the dropout rate of the three middle blocks of the bottleneck model from 0.5 to 0.2. Figure 7 illustrates the instance-diverse outputs generated by the sRD-GAN with the light residual dropout in inference mode, where all the synthetic COVID-19 CT images contain prominent findings of GGO that are generated in photorealistic quality. The observed patterns of synthetic GGO are diverse regarding the shapes and locations at which the features are manifested without affecting the structures of the chest and the regions of the lungs.

**Figure 8:**
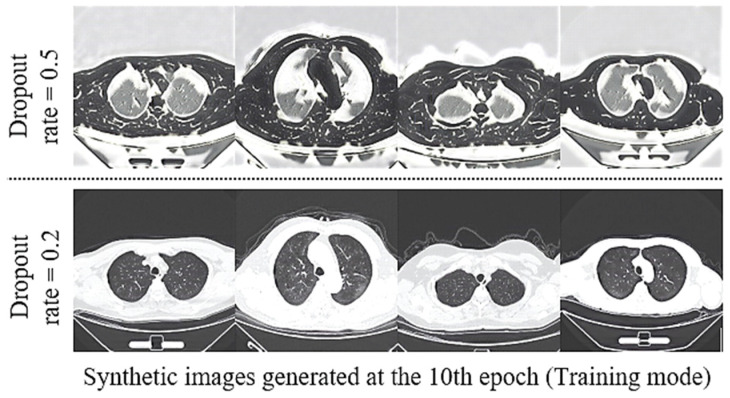
Comparison of the synthetic outputs generated from sRD-GAN trained with six RD-blocks with a dropout rate of 0.5 and a reduced dropout rate of 0.2 for the three middle RD-blocks.

The improvement contributed by the sRD-GAN trained with a light residual dropout setting is illustrated in Figure 8, where the model can generate plausible images at the early stage of the training. On the contrary, the counterpart model that is trained with a fixed dropout rate of 0.5 is still in a state of instability due to the larger magnitude of latent stochasticity. Based on these observations, the images suffer massive distortion, where the intensity levels that describe the chest and the background structures are disfigured to a significant degree.

**Figure 9:**
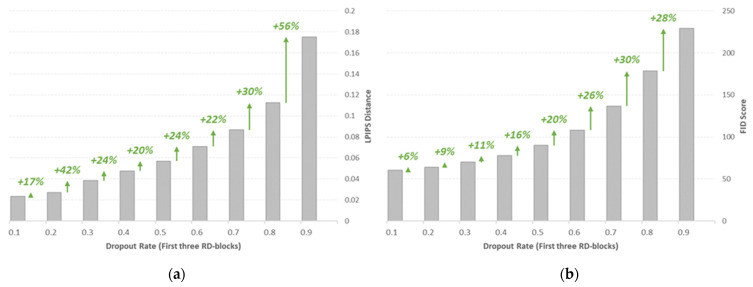
(**a**) LPIPS and (**b**) FID metrics of the synthetic images generated with different dropout rates in the inference mode. Performance metrics are summarized in Table S1.

The pre-trained sRD-GAN with the light residual dropout setting was adopted directly in the inference mode, where nine dropout rates were considered, ranging from 0.1 (lowest rate) to 0.9 (highest rate). The dropout rates were applied only to the first three RD-blocks of the bottleneck model, and the dropout rate of the remaining residual blocks was fixed at 0.5. Figure 9 shows the LPIPS and FID metrics of the synthetic images generated with varying combinations of dropout rates. Specifically, both metrics show an exponentially increasing trend as the dropout rate increases. Notably, the most significant increment of LPIPS distance is identified from the dropout rate of 0.8 to 0.9, of 56%. A similar scenario is observed for the FID score, with the largest FID score of 229.11, signifying the poorest image quality with a 0.9 dropout rate. The negative correlation between the image instance diversity and the perceptual quality observed in this experiment supports the previous claim that specifies the trade-off between both qualities due to the expanded space of latent stochasticity.

#### 3.2.2. Single RD Activation at Different Latent Depths

High dimensionality processes geometrical structures such as lines and contours in deep learning, while low dimensionality processes more abstract structures. Since the latent stochasticity is induced directly in the latent space of the image transformation network, the correlation between perceptual image diversity and dimensionality is likely to be associated. Therefore, the residual dropout at different dimensionalities of the image transformation network can be further investigated with a single RD-activation at different orders of the residual blocks that represent the depth of the latent space. In this experiment, only one residual dropout was activated at nine different residual blocks of the image transformation network at a time. Thereby, nine inference configurations were set up since there are nine residual blocks in the image transformation network. The dropout rate was fixed at 0.5 for the RD-blocks. Similar to the experiment discussed in Section 3.2.1, the pre-trained sRD-GAN with the light residual dropout setting was adopted.

**Figure 10:**
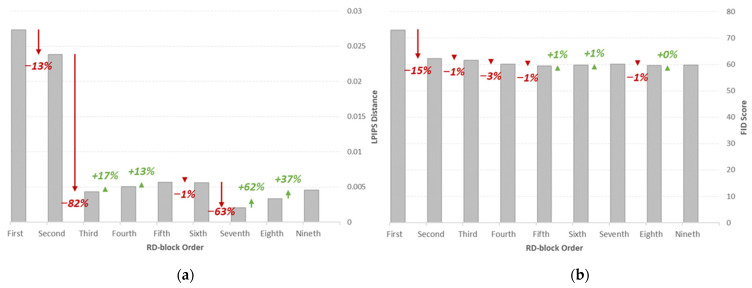
(**a**) LPIPS and (**b**) FID metrics of the synthetic images generated at different orders (single RD activation). Performance metrics are also summarized in Table S1.

Figure 10 shows the LPIPS and FID metrics of the synthetic images generated with a single residual dropout regularization layer at nine different residual blocks of the image transformation network. It is shown that the addition of the residual dropout at the first and the second residual block achieves significantly larger LPIPS distances compared to the other variations, with the highest of 0.0273 for the first RD-block and 0.0238 for the second RD-block, followed by a drastic decrease of 82% for the third RD-block. For perceptual quality, the synthetic images generated at the first RD-block obtain the highest FID score of 73.091, which is relatively higher compared to the other experiment variations. This behavior highlights the impact of introducing latent space at high dimensionality, which has the most significant impact on affecting the structural representation of the images.

The inconsistent trend of the FID and the LPIPS metrics of the images generated after the third order suggests that the orders of the single residual dropout at lower dimensionalities do not contribute any significant differences to the magnitude of stochasticity. This scenario is likely caused by the deeper latent space that processes more abstract information, which is usually not translatable from the human cortex of visualization.

#### 3.2.3. Sequential Stacked RD Activation

The stack of RD-blocks is a sequential accumulation of residual dropout regularization layers of the sRD mechanism. However, unlike the experiment in Section 3.1, this experiment is performed only in inference mode. The model is adopted directly from the pre-trained sRD-GAN with light residual dropout, similar to Section 3.2.1 and Section 3.2.2. In inference mode, the number of RD-blocks is increased one at a time, from one block to nine blocks. Similarly, no model training is required as the images are generated in inference mode only.

**Figure 11:**
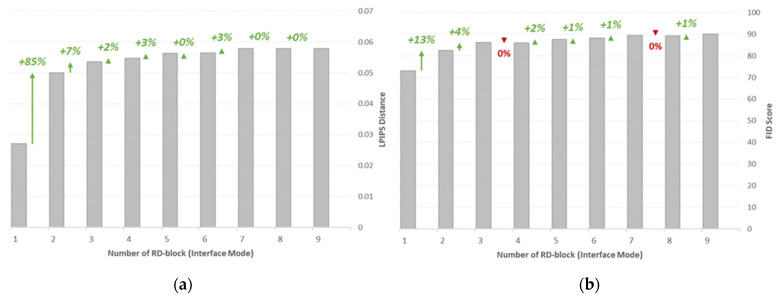
(**a**) LPIPS and (**b**) FID metrics of the synthetic images generated with varying numbers of RD-blocks in inference mode. Performance metrics are also summarized in Table S1.

The LPIPS and FID metrics of the synthetic images generated with different numbers of RD-blocks in the inference mode is demonstrated in Figure 11, where a sharp increase in LPIPS distance is observed from a single RD-block to two RD-blocks, and the size of the increment is smaller for the larger number of RD-blocks. This result is consistent with the previous suggestion, where the significance of the stochasticity induced at lower dimensions is too small to make any significant contribution to perceptually visible image dissimilarities. For the same reason, the difference between the FID score of the images generated with more than two RD-blocks is also insignificant. Nevertheless, the largest FID score identified is 89.46 for nine RD-blocks, and the lowest is 73.09 for one RD-block. This is expected as the larger number of RD-blocks will increase the capacity of latent stochasticity.

### 3.3. Qualitative Assessment

**Figure 12:**
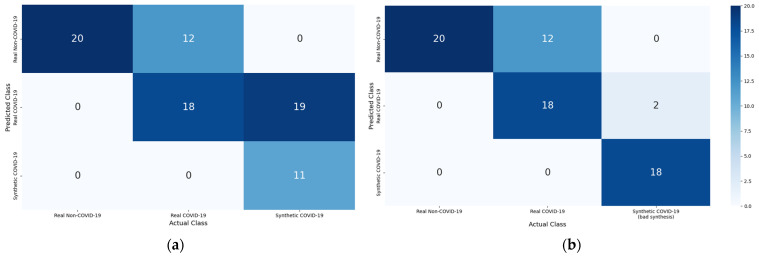
Confusion matrix of the Visual Turing Test: (**a**) 30 images of good image synthesis, (**b**) 20 images of bad image synthesis, compared to real COVID-19 and non-COVID-19 CT images.

The synthetic COVID-19 CT images achieved encouraging results based on the Visual Turing Test, with 63.33% of the synthetic images identified to contain clinical features consistent with the real COVID-19 CT images. The result of the Turing Test is included in Figure 12.

**Figure 13:**
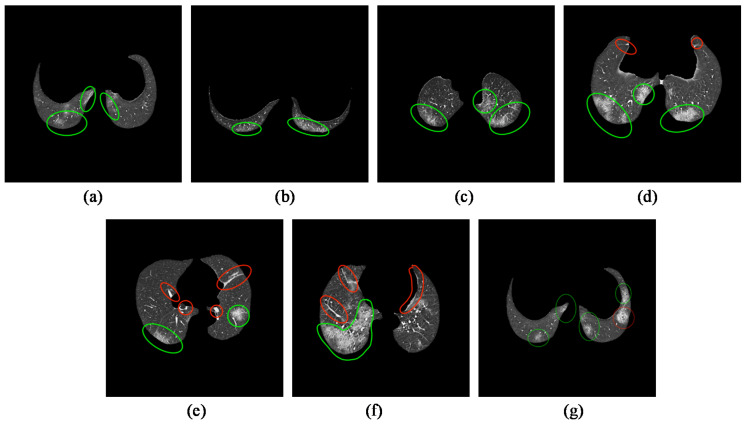
(**a**–**g**) Samples of synthetic COVID-19 CT images examined by an experienced radiologist. The green annotations indicate findings of GGO, whereas red annotations indicate irrelevant features of GGO.

Figure 13 illustrates samples of synthetic COVID-19 CT images, which were claimed to contain radiological findings of GGO when examined by a radiologist. This result suggests that sRD-GAN can generate photorealistic quality CT images with diverse patterns of GGO in different shapes of the lungs.

**Figure 14:**
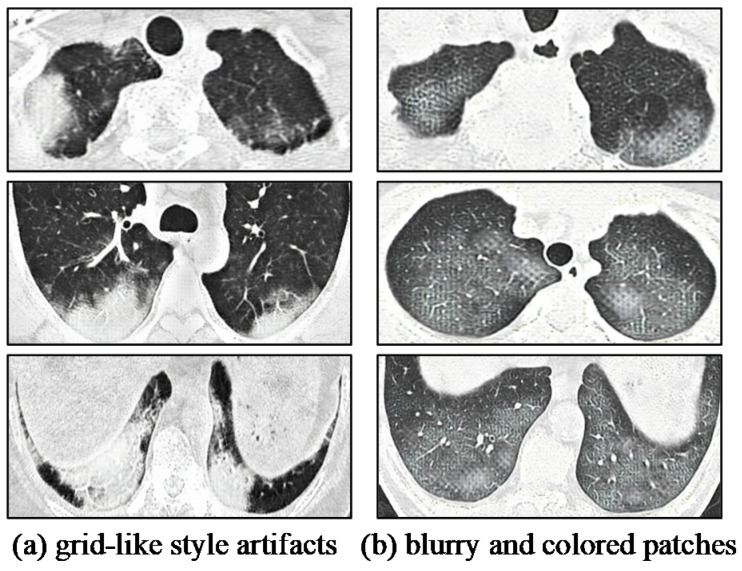
(**a**,**b**) Examples of style-artifacts manifested on synthetic COVID-19 CT images. These samples are generated from other GAN baselines.

Among the twenty synthetic COVID-19 CT images with style artifacts, only two images were examined as real COVID-19 CT, which signifies the repercussion of style artifacts, which is detrimental to the realistic representation of the synthetic images. An illustration of the style artifacts’ post-image translation is included in Figure 14. These style-artifacts are caused by ineffective learning due to the expanded space of stochasticity. In addition, there is no synthetic COVID-19 image classified as non-COVID-19 in the test, and this demonstrates the consistency of sRD-GAN in generating synthetic features on the output images; that is, the synthetic images contain anomalies that may not be related to COVID-19.

### 3.4. Effective Noise Reduction via Pixel Consistency Loss

#### 3.4.1. Adaptive Pixel Consistency Loss

GAN-based image translation models are hard to train and even more challenging when the image domain is specific and new. The successful synthesization of deep counterfeit COVID-19 CT images that are hardly distinguishable from real COVID-19 CT images in this study is attributed to a dynamic loss function that penalizes pixel-level inconsistency.

This section discusses the effectiveness of the proposed adaptive pixel consistency loss in enhancing the perceptual quality of the synthetic images by effective reduction in noises. The proposed adaptive setting of the pixel consistency loss is compared to its fixed setting counterparts, defined by a constant variable that controls the magnitude of the loss. Specifically, the lowest value was defined at 10, followed by 20 and 30 as the largest. For comparison purposes, the other GAN baselines, including the CycleGAN and GAN, were modified with the proposed pixel consistency losses. Since the other GAN baselines do not generate diverse outputs, the images were only generated from the training mode for fair comparisons. For the same reason, the LPIPS metric computed between the synthetic output batch and the input batch was used to evaluate the significance of synthetic GGO patterns manifested on the output image, and was not used to be confused with the LPIPS metric computed between the synthetic outputs to evaluate instance diversity.

Table 2 summarizes the performance metrics of the sRD-GAN and the GAN baselines. Overall, it is identified that large pixel consistency weight reduces the perceptual significance of the synthetic features represented by low LPIPS distances and achieves good perceptual quality represented by low FID score, as noticed in sRD-GAN and CycleGAN. In contrast, small pixel consistency weight results in high LPIPS distance and high FID score. This scenario describes the negative relationship between the significance of synthetic features and the quality of the images. However, this relation does not apply to GAN because the impact of the pixel consistency loss is insignificant compared to the unconstrained noise generated by the GAN that is trained without any mapping constraint. This is proven by the inconsistent performance of the images generated by the GAN using different settings of pixel consistency loss, as observed in Table 2.

**Figure 15:**
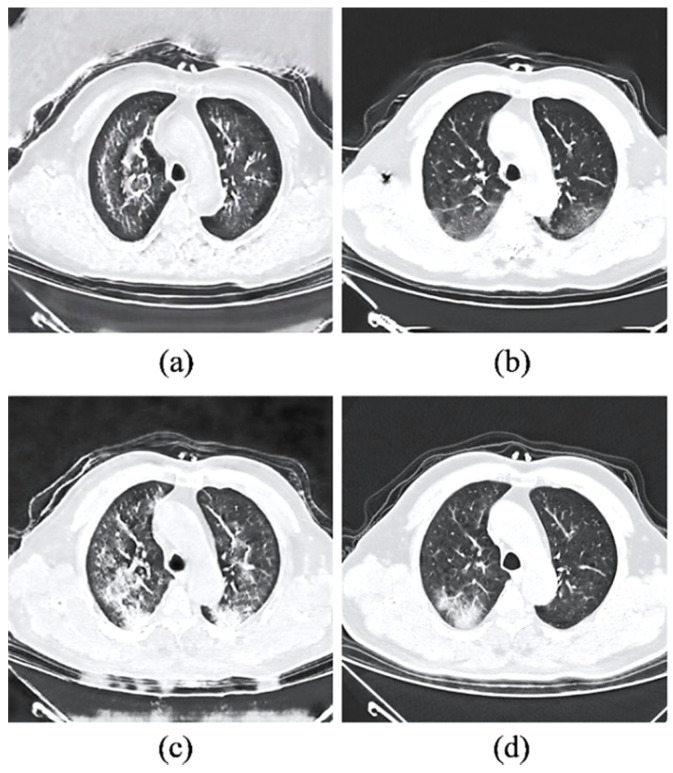
Examples of synthetic COVID-19 images generated from (**a**) GAN (without both cycle and pixel consistency loss, (**b**) GAN + pixel consistency, (**c**) CycleGAN (without pixel consistency), and (**d**) CycleGAN + pixel consistency.

The significance of the adaptive setting of the pixel consistency loss is justified by the upper-bounded performances achieved by the one-to-one CycleGAN and CycleGAN with the adaptive setting of pixel consistency loss compared to its constant setting. Notably, the adaptive setting of both GAN baselines achieved relatively higher LPIPS distance without sacrificing the perceptual quality of the images. Figure 15 shows the effectiveness in noise-artifact reduction based on the image generated with adaptive pixel consistency constraint. In particular, the white spots and the blurry lines on the background structure of the CT image illustrated in Figure 15a appeared to be reduced drastically compared to Figure 15b.

#### 3.4.2. Difference between Pixel and Cycle Consistency

While both pixel consistency loss and cycle consistency loss contributed to the perceptual quality enhancement of the synthetic images using the same mapping constraint strategy, it is observed that both losses behave differently, as observed from the output of the GAN baselines in Figure 15. Based on the comparison of the image generated with and without the respective mapping constraints, it is demonstrated that pixel consistency loss is more focused on noise reduction, and it cannot preserve high-frequency structural information. By comparison, cycle consistency loss utilized in CycleGAN and sRD-GAN is excellent in preserving structural information. However, cycle consistency alone fails to effectively reduce noise interference, which is visible in the background of the chest structure, as shown in Figure 15c.

### 3.5. Additional Analysis

#### 3.5.1. GradCAM Analysis

**Figure 16:**
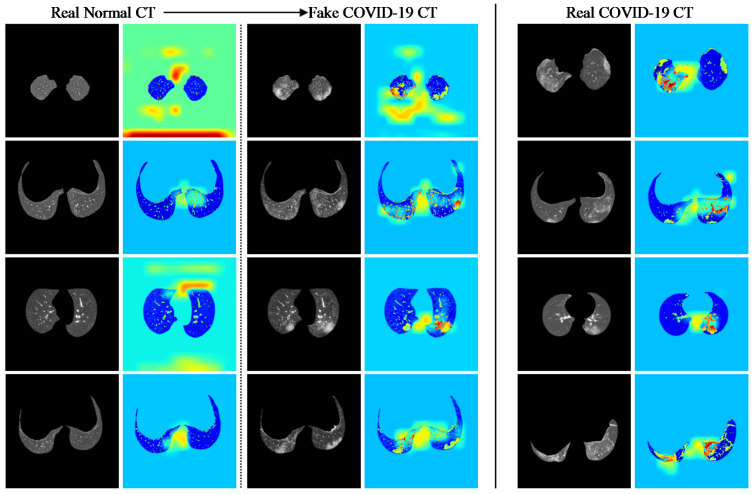
Grad-CAM of a pre-trained COVID-19 detection model on real non-COVID-19, real COVID-19, and synthetic COVID-19 CT images.

To further evaluate the information correlations between the real and synthetic COVID-19 CT data, the GradCAM responses of a pre-trained COVID-19 detection model [57] were generated using real and synthetic COVID-19 CT images, as shown in Figure 16. Furthermore, the shape of the lungs and the region of interest of GGO in both real and synthetic images were intentionally matched to the best effort to identify the differences in the GradCAM response between both set of images. In particular, non-COVID-19 CT shows very different responses compared to real and synthetic COVID-19 CT, where the heatmaps generated from the non-COVID-19 CT images mainly focus on the background rather than the chest structures. On the contrary, it is noticed that the heatmaps generated from both real and synthetic COVID-19 CT images are perceivably similar in different shapes of the chest structures. The similarity of the synthetic COVID-19 CT images with real COVID-19 CT images can be observed by similar heat map behavior generated on both distributions on approximately similar patterns of GGO and chest structures.

#### 3.5.2. Pixel Intensity Distributions and UMAP Analysis 

**Figure 17:**
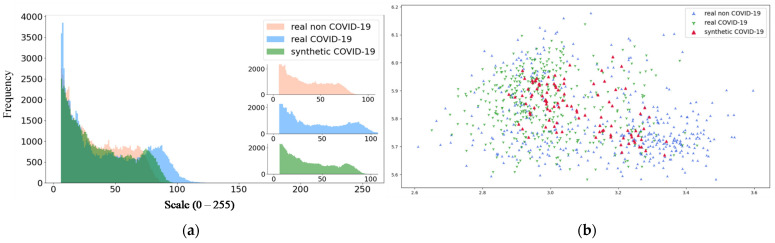
(**a**) Pixel distribution and (**b**) UMAP scatter plot of real COVID-19 real non-COVID-19, and synthetic COVID-19 CT images.

The similarity of the synthetic COVID-19 CT images with real COVID-19 CT images is validated by the intensity distribution of the images, as illustrated in Figure 17a, where the synthetic image distribution exerts a high degree of overlap with the distribution of the real COVID-19 CT. In addition, a UMAP [56] scatter plot is computed using three different types of distributions, as shown in Figure 17b. Each data point is computed from the output generated from the final convolution block of the previously trained COVID-19 detection model [57]. From the figure, it is shown that the synthetic instances are well blended with the real instances, where a large portion of the synthetic instances (denoted by red marks) reside around the real COVID-19 (denoted by green marks) and the minority with the real non-COVID-19 (denoted by blue marks). This is reasonable due to the high tolerance of texture artifacts and the high similarity of the image structure between COVID-19 and non-COVID-19 CT images, especially when features of GGO found within the region of the lungs are perceptually negligible.

#### 3.5.3. Performance on HUST-19 Dataset 

The performance of sRD-GAN (light residual dropout setting in training mode and 0.2 dropout rate in inference mode) was evaluated on the HUST-19 dataset [4], where 30 test sets were constructed from 3000 non-COVID-19 CT images. Therefore, each test set contained 100 randomly selected images from the entire HUST-19 dataset. Details about the dataset are presented in Section 2.1.

**Figure 18:**
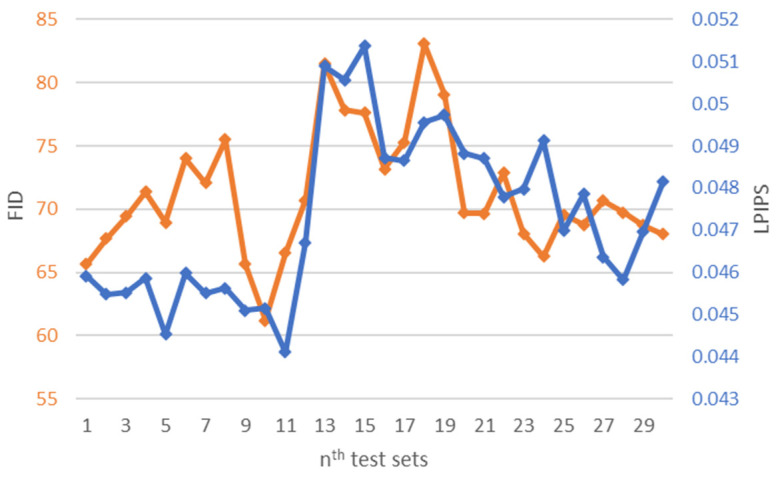
FID and LPIPS metrics of the synthetic COVID-19 CT images generated by sRD-GAN on the 30 test sets from the HUST-19 dataset [4]. Synthetic samples are included in Dataset S3.

The LPIPS and the FID metrics of the 15,000 synthetic COVID-19 CT images generated by sRD-GAN using the 30 test sets are illustrated in Figure 18. The testing protocol was similar to the preliminary experiment, where one input dataset generates five output datasets. The LPIPS and the FID metrics were computed using the average score of both measurements on the five output datasets. Based on Figure 18, the perceptual quality and instance diversity of the synthetic images exert a negative correlation consistent with the presented results, where an increase in LPIPS results in a decrease in image quality (higher FID scores). However, it is also observed that such a relationship is not followed strictly in some test sets due to the stochastic nature of GAN algorithms during the mapping process, and imposes a higher impact on the image quality than the effect of the sRD regularization. This also explains the differences in the metrics between the test sets generated using the same generator model, which are caused by the distribution bias that generates different mapping results on different inputs.

### 3.6. Benchmarking with Existing GANs

The sRD-GAN framework for non-COVID19-to-COVID19 CT I2I translation was compared to the existing GAN baselines on the same translation task, including the GAN [52], CycleGAN [45], and one-to-one CycleGAN [36]. For sRD-GAN, the one trained with six RD-blocks at the reduced dropout rate of 0.2 (discussed in Section 3.2.1, Section 3.2.2 and Section 3.2.3) was selected to benchmark against the images generated from other GAN baselines. Details of the neural network architectures of the generator and discriminator models can be found in [46,52], respectively. The training and testing procedures of the GAN baselines were similar to those of sRD-GAN for a fair comparison, except for the underlying network architectures and loss functions for each GAN algorithm.

**Figure 19:**
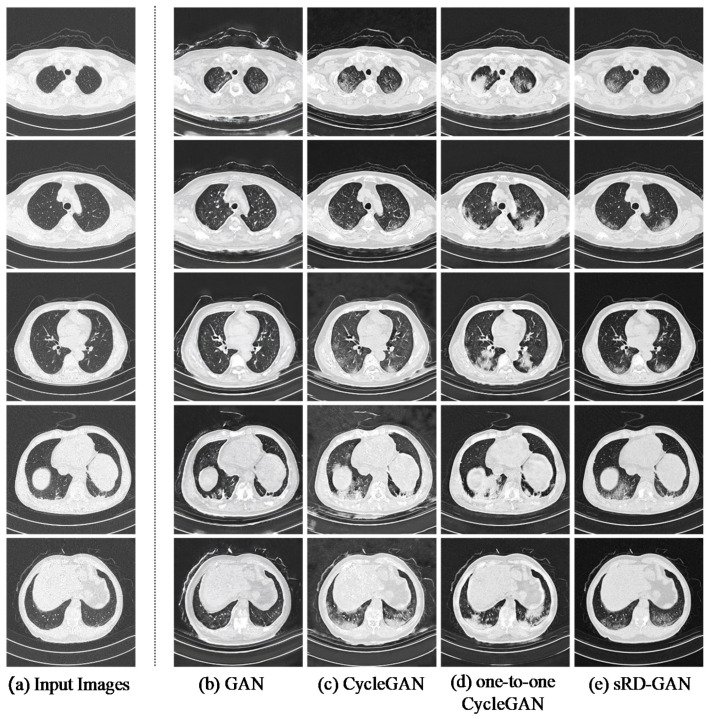
(**a**) Real COVID-19 CT images, (**b**–**e**) example of synthetic images generated by the proposed sRD-GAN and other GAN baselines. The synthetic images generated from GAN and CycleGAN contain a suboptimal realistic representation of the synthetic instances with large magnitude of noise artifacts.

Based on Table 3, the images generated by the proposed sRD-GAN achieve superior results compared to other GAN baselines. Remarkably, the FID score achieved by sRD-GAN is approximately threefold compared to the GAN and twofold compared to CycleGAN and one-to-one CycleGAN. For the significance of synthetic GGO features, it is shown that the images generated from the sRD-GAN manifested the least significant magnitude of synthetic features compared to other GAN baselines, which is likely due to the impact of noise reduction facilitated by the adaptive pixel consistency constraint. This is because the synthetic GGO features are essentially noises that contain structural meaning that could be understood by perceptual observation. Nonetheless, the relatively smaller LPIPS distance does not affect the overall visibility of the features of GGO in synthetic COVID-19 CT images. Examples of the synthetic COVID-19 CT images generated by the GAN models are illustrated in Figure 19.

In addition, it is found that the synthetic images generated by the GAN model suffer significant noise interference and a large magnitude of information distortion. The background noises appeared as large spots that are greyish-white in color. Moreover, the synthetic GGO features are presented in blurry conditions, with hazy lines and border structures. By comparison, images generated by the CycleGAN model improve drastically in terms of perceptual quality, which is also indicated by the lower FID score. The improvement is attributed to the bidirectional mapping mechanism that enforces a cycle-consistency constraint between the image domains at the cost of doubling the training duration to ~106.67 h, compared to ~58.67 h with GAN. Since sRD-GAN and one-to-one CycleGAN are trained with cycle-consistency loss, the training duration is similar to that of the original CycleGAN, at ~106.67 h, despite only one generator model being used.

However, it is also noted that CycleGAN is ineffective in noise reduction, as suggested in Section 3.4.2, in which the considerably significant noise artifacts are frequently visible in the background of the structure of the chest region. On the contrary, it is shown that images generated by the one-to-one CycleGAN achieved better image quality than those of CycleGAN using a single image translator model. The improvement could be due to the stronger constraint exerted by the self-inverse property of the generator model, which effectively reduces the possible mapping space [36].

### 3.7. External Validation on Different Clinical Cases

The proposed sRD-GAN framework was adapted to two different clinical cases to validate the reproducibility of the proposed method in facilitating instance diversity and perceptual realism. For this purpose, the sRD-GAN framework was extended to: (1) CT images of community-acquired pneumonia (CAP) and (2) X-ray images of COVID-19. The model architecture, hyperparameters, and training options were similar to the setting of sRD-GAN for fair comparison purposes. The number of training images and the parameters of the sRD-GAN were not modified from the original setting of the experiment when applied in the two external clinical cases.

**Figure 20:**
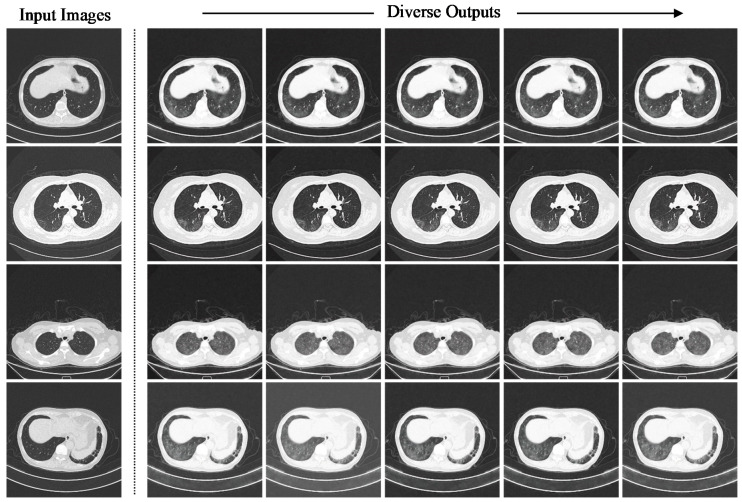
Examples of instance-diverse CAP CT images generated from sRD-GAN.

**Figure 21:**
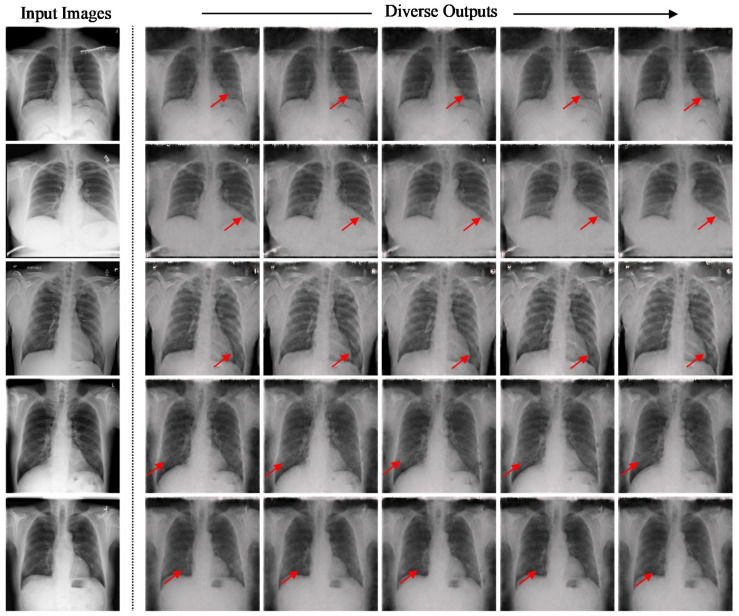
Examples of instance-diverse COVID-19 X-ray images generated from the sRD-GAN framework. The red arrows point to the region where the style attributes generated on the output images are different.

Based on observation, the synthetic images generated by the sRD-GAN in both clinical cases achieved a comparable result to the above-discussed COVID-19 CT image synthesization task, with good perceptual quality and perceptually visible instance diversity. This suggests that the proposed sRD-GAN framework can be easily extended to similar medical imaging applications, including different imaging modalities and diseases. However, it was observed that the perceivability of the instance diversity and the feature diversity for both clinical cases were relatively less significant compared to COVID-19 CT images, which is likely due to differences in information diversity and distribution shifts. Figure 20 and Figure 21 show examples of synthetic CAP CT and COVID-19 X-ray images, respectively. The red arrows in Figure 21 indicate the regions in which the instance diversity can be observed. Other samples are available in Dataset S4 and Dataset S5, respectively.

## 4. Discussion

### 4.1. Key Findings

A COVID-19 CT image I2I translation GAN framework named sRD-GAN is presented. The empirical investigations of various designs of the sRD regularization, the adaptive pixel consistency loss, and the performance benchmarking between existing GAN baselines can be summarized as follow:

1. Instance diversity. The main contribution of the novel sRD-GAN is the ability to facilitate perceptually visible instance diversity using a simple regularization-based strategy that is highly generalizable across GAN-based algorithms and without relying on auxiliary conditions. In this study, the experiment result suggests that sRD regularization can facilitate sufficient latent space stochasticity to induce a significant perceptual difference between the instance-diverse outputs. The in-depth investigation of the sRD mechanism from different perspectives reveals the positive correlation between the number of RD-blocks, dropout rate, and latent depths with the magnitude of instance diversity. Specifically, larger numbers of RD-blocks and larger dropout rates can enlarge the space of stochasticity and ultimately lead to increased instance diversity at the cost of perceptual quality degradation. In addition, the stochasticity induced at higher dimensionality can cause enormous structural changes, which lead to larger amplification of latent stochasticity compared to lower-dimensional latent spaces.
2. Perceptual realism and quality. While the perceptual quality can be evaluated using the standard FID metric, the actual reality of the synthetic COVID-19 remains challenging due to the domain knowledge requirement. With the help of an experienced radiologist, the Visual Turing Test reveals a promising result achieved by the synthetic images for generating radiography findings of GGO, which is consistent with the real COVID-19 CT images. Furthermore, exhaustive experiments demonstrate the consistent adversary correlation between image diversity and perceptual quality due to the underlying property of the stacked residual dropout, which induces latent space stochasticity and simultaneously encourages a more unconstrained space of image mapping. Thereby, a larger magnitude of stochasticity can generate additional noise artifacts that could be detrimental to the overall perceptual quality and realism of the images. The impact of the adversarial relationship between perceptual quality and image diversity is addressed by a reduced dropout rate at higher dimensional latent spaces. As a result, drastic improvement in the perceptual quality was noticed without affecting the significance of synthetic features generated on the output images. Furthermore, the sRD-GAN also demonstrated superior performance in terms of perceptual quality compared to other GAN baselines, where images generated from the GAN are distorted by a significant amount of noise artifacts. In contrast, the images generated from CycleGAN and one-to-one CycleGAN models failed to effectively eliminate the noise artifacts.
3. Effective noise reduction. The comparison between the images generated with and without pixel consistency and cycle consistency reveals the distinctive differences between the consistency losses in the image translation task. In particular, the proposed adaptive pixel consistency loss demonstrated superior performance in reducing the noise artifacts of the synthetic images. The effectiveness of the noise reduction is due to the strong connection enforced by the pixel consistency loss, which encourages the output image to be similar to the input image. Moreover, the adaptive setting of the pixel consistency loss addresses the problem of the diminished magnitude of the translated GGO features caused by the pixel consistency constraint. A possible explanation is that the conditional weight updates of the loss are based on the generator’s performance. The superior performance of the adaptive setting of the pixel consistency and its high effectiveness in reducing noise artifacts is also demonstrated on other GAN baselines.

### 4.2. Failure in Image Synthesis and Limitations

Based on the observation of the synthetic COVID-19 CT images generated by different GANs in the study, it is found that some CT images consistently failed to generate synthetic features of GGO compared to the majority portion of the chest CT images in the dataset. While it is hard to explain this ambiguous response of the GANs towards a subset of input distributions, a conditional mode collapse caused by the input distribution gap would explain the outlined scenario. As such, training with more samples with diversified visual descriptors of the chest CT can reduce the impact of the conditional mode collapse, which reduces the probability of synthetization failure. Nonetheless, the number of failure translations identified in the test set based on the observation was small, and such failure only happened on a small group of images with unique shapes and patterns.

In addition, it is also impossible to generate any targeted output instance in the problem setting of unpaired I2I translation, including the exact size, location, pattern, density, and color components of the feature of interest. In other words, the sRD-GAN depends neither on predefined conditions nor auxiliary conditions in training and inference modes, which essentially preserve the stochastic properties of conventional unpaired image translation approaches. For this reason, there is no guarantee that only patterns of GGO are generated in the synthetic images; other irrelevant features, such as the noise artifacts, can possibly be generated along with the GGO features. However, this issue can be controversial since the other features are regarded as noises, and could be leveraged to improve the robustness of the diagnostic models when the synthetic images are used to supplement the training activities.

While the image translation problem of non-COVID-19-to-COVID-19 assumes a shared content latent space that overlaps the large portion of the invariant features of the images belonging to both image domains describing the geometrical structures of the chest CT images, the instance diversity facilitated by the sRD mechanism is only limited to fine-grained feature transfer, which only involves synthesization of small and detailed features. Therefore, adapting the proposed method in other applications that require a large magnitude of perceptual differences needs further investigation.

Finally, the metadata of the original CT images were not available in this study. They may be available upon request from the dataset sources.

## 5. Conclusions

The COVID-19 pandemic is a pertinent example in emphasizing the inevitable dependencies of modern ML techniques on the rapid acquisition of quality data, such that the data quantity is sufficiently large to train a ML model to make meaningful decisions when applied to real-world problems. Synthetic data is one of the most practical applications of the field of ML, especially when the artificial instances generated on the synthetic images are indistinguishable from the real images, as demonstrated by the COVID-19 CT modality in this paper. As such, strategic utilization of synthetic data within the ML pipeline in combating infectious diseases such as COVID-19 can reduce the risk of virus exposure since the actual imaging procedure is no longer necessary for acquiring data for a wide range of AI-assisted clinical applications. As a result of the new regularization strategy, which does not require any non-trivial modification to the underlying neural network architecture, studies of the effectiveness of the proposed method on other diseases, and its contribution to the more recent topics of AI, such as zero-shot or few-shot learning, may be interesting future research works.

## Figures and Tables

**Table 1:** Summary of datasets and descriptions.

Source	Modality	Class	Quantity
[4]	CT	COVID-19	100 patients
		Non-COVID-19	200 patients
[4]	CT	Non-COVID-19	9575 images
[48]	CT	COVID-19	416 patients
		CAP	412 patients
[49]	X-ray	COVID-19	3216 images
		Non-COVID-19	10,192 images

**Table 2:** Performance of GAN models with different pixel consistency loss settings.

Model	LPIPS	FID
sRD-GAN		
Adaptive	0.1254	38.9390
Constant = 10	0.1265	62.2360
Constant = 20	0.1186	43.3440
Constant = 30	0.0825	37.7170
CycleGAN		
Adaptive	0.2862	116.8220
Constant = 10	0.2776	125.7760
Constant = 20	0.2553	79.0550
Constant = 30	0.1973	63.4590
GAN		
Adaptive	0.2244	110.0560
Constant = 10	0.2132	89.7050
Constant = 20	0.1879	91.8460
Constant = 30	0.2072	94.6160

**Table 3:** Performance metrics of the images generated from different GAN baselines.

Model	Training Duration	LPIPS (Significance of Features)	FID
sRD-GAN	~106.67 h	0.1370	58.6774
One-to-one CycleGAN	~106.67 h	0.1952	94.1130
CycleGAN	~106.67 h	0.3055	115.1420
GAN	~58.67 h	0.3905	157.1800

## Data Availability

Data is contained within the article or supplementary material. The data presented in this study will be made available.

## Supplementary Materials

The following supporting information can be downloaded at: https://zenodo.org/record/7325501#.Y3bnF31BzQw, Table S1: Summary of performance metrics for Section 3.2; Dataset S2: Instance-diverse synthetic COVID-19 CT images generated from the sRD-GAN with light residual dropout; Dataset S3: Synthetic COVID-19 CT images generated from the HUST-19 dataset; Dataset S4: Synthetic CAP CT images generated by sRD-GAN; Dataset S5: Synthetic COVID-19 X-ray images generated by sRD-GAN.

## Appendix A

Similar to [36], only a single generator model is used in the GAN framework. The generator model consists of three major components: two down-sampling blocks, nine residual connection blocks, and two up-sampling blocks. The input dimension of the generator model is fixed at 256 × 256 × 3 and is the same for output images.

The naming convention in Table A1 and Table A2 is referenced directly from Johnson et al.’s GitHub repository [52]. c7s1-k refers to a 7 × 7 convolution with k filters and stride of 1—normalization—activation. dk refers to a 3 × 3 convolution with k filters and stride of 2—normalization—activation. Rk denotes a residual block with two sub-blocks of 3 × 3 convolution with k filters and stride of 1. uk refers to a 3 × 3 transposed convolution with k filters and stride of 0.5. ReLU activation is used for all layers except in the output block, which uses the tanh activation. Instance normalization is used in all normalization layers. Reflection padding is used before convolution layers in every block except in down-sampling and up-sampling blocks.

**Table A1:** Architecture of generator model.

Block	Components	Output Shape
Input Block	c7s1-64	256, 256, 64
Down-sampling Block 1	d128	128, 128, 128
Down-sampling Block 2	d256	64, 64, 256
Residual Block 1	R256	64, 64, 256
Residual Block 2	R256	64, 64, 256
Residual Block 3	R256	64, 64, 256
Residual Block 4	R256	64, 64, 256
Residual Block 5	R256	64, 64, 256
Residual Block 6	R256	64, 64, 256
Residual Block 7	R256	64, 64, 256
Residual Block 8	R256	64, 64, 256
Residual Block 9	R256	64, 64, 256
Up-sampling Block 1	u128	128, 128, 128
Up-sampling Block 2	u64	256, 256, 64
Output Block	c7s1-3	256, 256, 3

The discriminator is referenced from the pix2pix architecture [46]. PatchGAN maps an input distribution to the variable size of N×N arrays instead of generating outputs of a single scalar output. Therefore, each value of the squared activation outputs represents a likelihood probability that the input image is real. The value chosen for N in this study was fixed as 70, which is based on the recommendation of the authors of the paper. Details of the model can be found in the original paper of pix2pix GAN [46].

**Table A2:** Architecture of the discriminator model.

Block	Components	Output Shape
Convolution Block 1	C64	128, 128, 64
Convolution Block 2	C128	64, 64, 128
Convolution Block 3	C256	32, 32, 256
Convolution Block 4	C512	16, 16, 512
Output Block		

Ck refers to a 4 × 4 convolution with k filters and stride of 2—normalization—activation. LeakyReLU, with a slope of 0.2, is used for activation, and instance normalization is used in all convolution blocks except Convolution Block 1. The output block is a one-layer 4 × 4 convolution with one filter to produce a single dimension output.
